# STAT6 induces expression of Gas6 in macrophages to clear apoptotic neutrophils and resolve inflammation

**DOI:** 10.1073/pnas.1821601116

**Published:** 2019-07-30

**Authors:** Saroj Nepal, Chinnaswamy Tiruppathi, Yoshikazu Tsukasaki, Joseph Farahany, Manish Mittal, Jalees Rehman, Darwin J. Prockop, Asrar B. Malik

**Affiliations:** ^a^Department of Pharmacology, University of Illinois College of Medicine, Chicago, IL 60612;; ^b^Center for Lung and Vascular Biology, University of Illinois College of Medicine, Chicago, IL 60612;; ^c^Institute for Regenerative Medicine, College of Medicine, Health Science Center, Texas A & M University, Bryan, TX 77807

**Keywords:** macrophage, STAT6, Gas6

## Abstract

Efferocytosis of apoptotic neutrophils (PMNs) by alveolar macrophages (AMФs) is vital for resolution of inflammation and tissue injury. Here, we investigated the role of AMФ polarization and expression of the efferocytic ligand Gas6 in restoring homeostasis. In the murine model of lipopolysaccharide (LPS)-induced acute lung injury (ALI), we observed augmented temporal generation of cytokines IL-4 and TSG6 in bronchoalveolar fluid (BALF). Interestingly, we also observed increased expression of antiinflammatory markers consistent with a phenotype shift in AMФs. In particular, AMФs expressed the efferocytic ligand Gas6. In vitro priming of bone marrow–derived macrophages (BMMФs) with IL-4 or TSG6 also induced MФ transition and expression of Gas6. TSG6- or IL-4–primed BMMФs induced efferocytosis of apoptotic PMNs compared with control BMMФs. Adoptive transfer of TSG6- or IL-4–primed BMMФs i.t. into LPS-challenged mice more rapidly and effectively cleared PMNs in lungs compared with control BMMФs. We demonstrated that expression of Gas6 during AMФ transition was due to activation of the transcription factor signal transducer and activator of transcription-6 (STAT6) downstream of IL-4 or TSG6 signaling. Adoptive transfer of Gas6-depleted BMMФs failed to clear PMNs in lungs following LPS challenge and mice showed severely defective resolution of lung injury. Thus, activation of STAT6-mediated Gas6 expression during macrophage phenotype transition resulting in efferocytosis of PMNs plays a crucial role in the resolution of inflammatory lung injury.

## Significance

Clearance of apoptotic neutrophils by alveolar macrophages (AMФs) is critical for the resolution of acute lung injury (ALI). Here, we demonstrated that induction of the M1 phenotype in MФs activates the transcription factor STAT6 and thereby promotes resolution of lung injury by the increased expression of Gas6, the ligand for efferocytosis. Therefore, targeting the STAT6 activation pathway in AMФs and Gas6 may be a potential therapeutic strategy for resolution of inflammatory lung injury.

Alveolar macrophages (AMФs) are essential for recognition and clearance of pathogens from the airways, initiation of host defense, and tissue repair (1). During resolution of lung injury, AMФs clear apoptotic neutrophils (PMNs), tissue debris, and bacteria from the alveolar space through a tightly coordinated process known as efferocytosis (2, 3). Efferocytosis is critical for resolution of lung injury since impaired clearance of apoptotic PMNs by AMФs leads to continued inflammation and injury (1, 4). Efferocytosis is a well-conserved process as evident from the observation that IL-13 secreted by Treg cells resolves lung inflammation through activating MФ-mediated efferocytosis (5).

A distinct family of tyrosine kinase receptors known as TAM receptors, which comprise Tyro3, Axl, and Mer proteins, are essential for efferocytosis mediated by MФs (6, 7). MФs also generate the ligand growth arrest specific-6 (Gas6), which bridges MФ-expressed TAM receptors to the phosphatidylserine on the surface of apoptotic cells to trigger efferocytosis (8). The inflammatory response is associated with inflammatory MФ phenotype M1, which secretes proinflammatory cytokines, whereas resolution of inflammation is associated with MФ M2 phenotype shift, with MФs expressing antiinflammatory M2 markers (e.g., arginase-1 and CD206) (9, 10). The signaling events mediating up-regulation of efferocytotic components during MФ phenotype shift are not well understood. IL-4 and IL-13 generated by MФs promote MФ phenotype transition via activation of the transcription factor STAT6 (11–13). We showed that MФ secretes the protein TNF-α-stimulated gene-6 (TSG6), which is also essential for MФ phenotype transition; further, it promotes the resolution of sepsis-induced acute lung injury (14). To elucidate mechanisms of efferocytosis activated during MФ phenotype transition, we focused on the role of IL-4 and TSG6 signaling. We observed that both TSG6 and IL-4 induced increased STAT6-dependent expression of the efferocytosis ligand Gas6 in MФs and that Gas6 expression was required for the clearance of apoptotic PMNs. Furthermore, our results showed that adoptive transfer of TSG6- or IL-4–primed MФs i.t. into lipopolysaccharide (LPS)-challenged mice rapidly and effectively clears PMNs, raising the prospect of cell-based therapy in resolving inflammatory lung injury.

## Results

### MФ Phenotype Transition Promotes Efferocytosis in Alveoli.

**Fig. 1.**
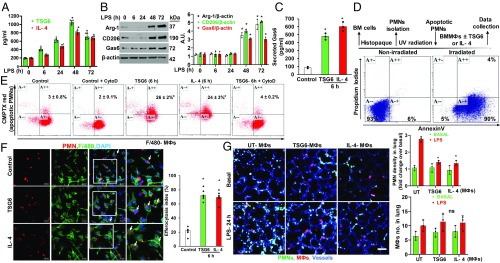
MФ phenotype transition promotes efferocytosis. (*A*) BALF collected from mice challenged with LPS for the indicated time points was used for the measurement of TSG6 and IL-4 by ELISA. *n* = 6 mice per time point. *P < 0.05 vs. basal. (*B*) Immunoblots showing arginase-1, CD206, and Gas6 expression in AMФs isolated from BALF from mice challenged with LPS for the indicated time points. Results shown are representative blots with β-actin as a loading control. *n* = 6 mice per time point. *P < 0.05 vs. basal. (*C*) Gas6 secreted in BMMФ culture media was measured by ELISA. Results shown are mean ± SD of three experiments. *P < 0.001 vs. control. (*D*) Scheme of experimental protocol for PMN isolation and apoptosis induction. Apoptosis induction was analyzed by flow cytometry after staining with FITC Annexin V and Propidium Iodide. More than 90% of PMNs were apoptotic (*Lower-* and *Upper*-*right quadrants*) in irradiated group. (*E* and *F*) Assessment of efferocytosis by BMMФs in vitro. (*E*) BMMФs treated with TSG6 (400 ng/mL) or IL-4 (100 ng/mL) for the indicated time points were overlaid for 2 h with apoptotic PMNs labeled with CellTracker CMTPX red dye in the presence or absence of cytochalasin D (2 µM, 2 h). Then the cells were fixed in 1% PFA and analyzed by flow cytometry. BMMФ populations positive for both F4/80 and CMTPX PMNs were considered to be the BMMФs engulfing apoptotic PMNs (*Upper*-*right quadrant*). Results shown are from separate experiments. *P < 0.05 vs. control. (*F*) same as *E* with confocal analysis to assess efferocytosis by BMMФs. F4/80 antibody BMMФs in green; apoptotic PMNs in red. Quantified data from three different experiments are shown in bar graph (*Lower right*); *P < 0.05 vs. control. (*G*) In vivo imaging of PMNs in alveolar space and lung microvessels using high-resolution 2-photon excitation microscopy. BMMФs were stimulated with TSG6 (400 ng/mL) or IL-4 (100 ng/mL) for 6 h and then i.t.-instilled into mice (2 × 10^6^ cells/mouse). Animals simultaneously received LPS (10 mg/kg i.p.) and BMMФs i.t. At 24 h after LPS challenge and BMMФs i.t., 2-photon images were collected as described in *Materials and Methods*. (Scale bar, 50 μm.) Quantitative analysis of PMN density in lung (*n* = 30–33 for each bar of wild type) and number of MФs in alveolar space (*n* = 5, *Right*). Alveoli were outlined and fluorescent intensities of PMNs were quantified; value of the basal condition in wild-type mice with unstimulated BMMФs was normalized as 1 (*Right*). *P < 0.001 vs. basal level with unstimulated BMMФs.

To determine whether AMФ phenotype shift occurs during resolution of endotoxin-induced inflammatory lung injury, we injected LPS (10 mg/kg i.p.) into wild-type (WT) mice. At different time points, we analyzed the concentrations of IL-4 and TSG6 in bronchoalveolar lavage fluid (BALF). We found significantly increased concentrations of IL-4 and TSG6 in BALF at 48 h after LPS challenge compared with controls (Fig. 1*A*). We also observed that AMФs obtained from BALF using anti-CD11b antibody at this time exhibited augmented expression of antiinflammatory M2 markers (arginase-1 and CD206) (Fig. 1*B*). Importantly, LPS challenge elicited augmented expression of Gas6 in the AMФs (Fig. 1*B*).

Since TSG6 and IL-4 are known to promote MФ phenotype transition, we next investigated the possibility that TSG6- or IL-4–primed bone marrow–derived macrophages (BMMФs) may functionally activate the efferocytosis of apoptotic PMNs. We observed that BMMФs exposed to either TSG6 or IL-4 elicited marked release of Gas6 in the medium (Fig. 1*C*). To study efferocytosis, we isolated PMNs from mice bone marrow and exposed them to UV radiation for apoptosis induction (15) (Fig. 1*D*). These PMNs were labeled with CellTracker CMPTX red dye. We observed that BMMФs primed with TSG6 or IL-4 effectively efferocytosed PMNs, as evident from the population of F4/80+ MФs engulfing apoptotic PMNs (Fig. 1*E*). To confirm whether our experimental conditions could distinguish between ingested apoptotic PMNs and PMNs merely bound on the MФ cell surface, we pretreated BMMФs with cytochalasin D [which inhibits actin rearrangements (16)] and then measured efferocytosis. Here we observed complete blockade of PMN ingestion in cytochalasin D–treated BMMФs (Fig. 1*E*), suggesting that the efferocytosis assay measured ingested and not cell surface–bound apoptotic PMNs. Confocal imaging analysis also showed enhanced PMN uptake by TSG6- or IL-4–primed BMMФs compared with untreated BMMФs (Fig. 1*F*). Next, using high-resolution 2-photon excitation microscopy, we determined efferocytosis in vivo by the control BMMФs and BMMФs primed with TSG6 or IL-4. Here, mice were challenged with LPS i.p. and BMMФs were instilled i.t. after having been either primed by TSG6 or IL-4 or not. In this experiment, blood vessels were labeled with anti-CD31 antibody (blue); PMNs, with anti-Ly6G antibody (green); and i.t. instilled BMMФs, with CellTracker CMPTX red dye. We observed that i.t. instillation of BMMФs primed with TSG6 or IL-4 effectively cleared the PMNs present in the airspace post-LPS compared with control BMMФs (Fig. 1*G*). Interestingly, we did not observe any difference in the number of adoptively transferred BMMФs present in lungs between the experimental groups (Fig. 1 *G*, *Right*).

### STAT6 Expression in Response to TSG6 or IL-4 Induces Gas6 Expression in MФs.

**Fig. 2.**
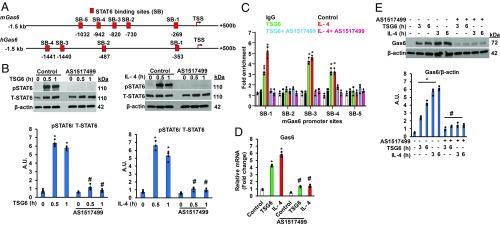
Transcription factor STAT6 activation promotes Gas6 expression in BMMФs. (*A*) Schematics of STAT6 binding sites in the 5′-regulatory regions of mouse (*m*) and human (*h*) genes encoding Gas6. (*B*) BMMФs were pretreated with STAT6 inhibitor AS1517499 (1 µM) for 1 h, followed by TSG6 (400 ng/mL; *Left*) or IL-4 (100 ng/mL; *Right*) stimulation for the indicated time points. Cell lysates were used for immunoblot analysis to determine STAT6 phosphorylation at Y641 and total STAT6 expression. Representative blots from three independent experiments are shown. *P < 0.05 vs. control. (*C*) BMMФs pretreated with AS1517499 (1 µM, 1 h) followed by either TSG6 (400 ng/mL) or IL-4 (100 ng/mL) stimulation for 1 h were used for ChIP. TSG6 or IL-4 induced STAT6 binding to three sites (SB-1, SB-3, and SB-4) in the *m*Gas6 promoter. Results are mean of three experiments normalized to those of input DNA and presented relative to control IgG values. *P < 0.05 vs. control. (*D*) BMMФs pretreated with AS1517499 were stimulated with either TSG6 (400 ng/mL) or IL-4 (100 ng/mL) for 1 h, and Gas6 mRNA expression was determined by RT-qPCR. *P < 0.05 vs. control; #*P* < 0.05 vs. TSG6- or IL-4–treated BMMФs without AS1517499 pretreatment. (*E*) Immunoblot analysis of Gas6 protein expression in BMMФs stimulated with TSG6 or IL-4 for the indicated time points with or without AS1517499 pretreatment. Results are representative of three independent experiments. *P < 0.05 vs. control; #*P* < 0.05 vs. TSG6- or IL-4–treated BMMФs without AS1517499 pretreatment.

The efferocytotic receptors (Tyro3, Axl, and Mer) constitutively expressed in MФs bind the secreted ligand Gas6, which bridges the MФ-expressed TAM receptors to the phosphatidylserine on the surface of apoptotic cells to trigger efferocytosis (8). We observed that expression of Gas6 in AMФs increased during the inflammation resolution phase after LPS challenge (Fig. 1*B*) and that IL-4 or TSG6 priming of BMMФs increased Gas6 expression (Figs. 1*C* and 2 *D* and *E*). To gain insights into transcriptional mechanisms of Gas6 expression during MФ phenotype transition, we analyzed the 5′-regulatory regions of both mouse (*m*) and human (*h*) genes encoding Gas6. We identified five putative binding sites for the transcription factor STAT6 upstream of the transcriptional start site (TSS) in the *m*Gas6 promoter (Fig. 2*A*) and four binding sites in the *h*Gas6 gene (Fig. 2*A*). We treated BMMФs with STAT6-specific inhibitor and measured TSG6- or IL-4–induced STAT6 phosphorylation, which induces STAT6 nuclear translocation and transcriptional activity (12). The STAT6 selective inhibitor (AS1517499) prevented TSG6- and IL-4–induced STAT6 phosphorylation (Fig. 2*B*). We next used chromatin immunoprecipitation (ChIP) assay to assess binding of STAT6 to consensus sites of the Gas6 promoter in response to IL-4 or TSG6 in BMMФs. We saw that either IL-4 or TSG6 challenge caused STAT6 binding to three sites (SB1, SB3, and SB4) in the proximal promoter region of the *m*Gas6 gene (Fig. 2*C*). Pharmacological inhibition of STAT6 prevented TSG6- and IL-4–induced Gas6 mRNA and protein expression in BMMФs (Fig. 2 *D* and *E*).

### Gas6 Expression in MФs Is Required for Efferocytosis.

**Fig. 3.**
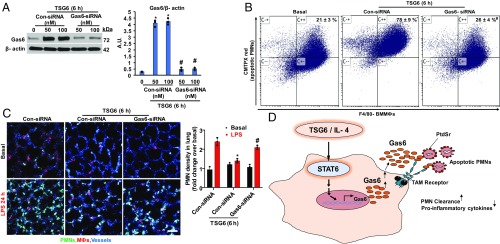
Gas6 expression in MФs is required for efferocytosis. (*A*) BMMФs transfected with control or Gas6 siRNA (sc-35451) were used to determine Gas6 protein expression by immunoblot. *P < 0.05 vs. control siRNA group. (*B*) BMMФs transfected with control or Gas6 siRNA were primed with TSG6 and then incubated with CMTP-labeled PMNs for 2 h. In vitro efferocytosis was analyzed by flow cytometry as in Fig. 1*E*. *P < 0.05 vs. basal; ^#^*P* < 0.05 vs. TSG6-treated control siRNA group. (*C*) BMMФs transfected with control or Gas6 siRNA were primed with TSG6 and adoptively transferred i.t. into mice challenged with LPS, followed by in vivo imaging of mouse lungs using high-resolution 2-photon excitation microscopy as in Fig. 1*G*. *P < 0.05 compared with control siRNA group without TSG6 stimulation. ^#^*P* < 0.05 vs. control siRNA BMMФs primed with TSG6. (*D*) Proposed signaling mechanisms of inflammation resolution induced by TSG6 or IL-4 reprogramming of MФs. TSG6- or IL-4–induced STAT6 activation promotes Gas6 expression in MФs. Once secreted, Gas6 links phosphatidylserine (PtdSr) on apoptotic PMNs to TAM receptor kinases expressed on MФs. This results in PMN clearance and suppression of proinflammatory cytokine production, thereby resolving lung injury.

To address the role of Gas6 in mediating efferocytosis, we suppressed Gas6 expression by transfecting BMMФs with Gas6 small interfering RNA (siRNA). We observed markedly reduced Gas6 protein expression compared with control siRNA-transfected cells (Fig. 3*A*). Next, we determined the effects of Gas6 knockdown on MФ efferocytosis in vitro and observed significantly reduced engulfment of PMNs compared with control TSG6-primed BMMФs (Fig. 3*B*). We also suppressed Gas6 expression using siRNA in MФs, and observed decreased MФ efferocytosis in vitro (*SI Appendix*, Fig. S1 *A* and *B*), further supporting the requisite role of Gas6 in the mechanism of efferocytosis in phenotype-shifted MФs. In addition, we determined the effects of Gas6 depletion in BMMФs in clearing lung airspace PMNs in vivo. In contrast to the active efferocytosis of airspace PMNs induced by instillation of TSG6-primed BMMФs (Fig. 1*G*), we found that instillation of Gas6-depleted BMMФs failed to clear PMNs (Fig. 3*C*). Based on these results, we propose a model for the mechanism of resolution of lung injury by MФs through activation of PMN efferocytosis following phenotype transition of MФs (Fig. 3*D*).

### TSG6-Primed BMMФs Resolve Inflammatory Lung Injury in Mice.

**Fig. 4.**
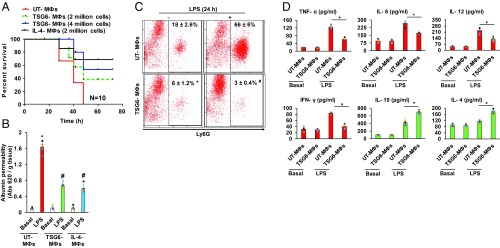
Adoptive transfer of TSG6-primed MФs via i.t. instillation suppresses LPS-induced lung injury. (*A*) Age- and weight-matched mice were challenged with a lethal dose of LPS (20 mg/kg i.p.) and i.t.-instilled with indicated number of TSG6- or IL-4–stimulated or unstimulated BMMФs. Mortality was monitored for 72 h. The mice instilled with TSG6- or IL-4–stimulated BMMФs (TSG6 MФs and IL-4 MФs) showed markedly increased survival compared with the unstimulated BMMФs (UT-MФs) group. *P < 0.05 vs. UT MФs group; *n* = 10 per group. (*B*) Lung vascular permeability (uptake of EBA) was assessed in LPS-challenged (10 mg/kg i.p.) mice concurrently instilled with UT or TSG6- or IL-4–stimulated MФs. The mice instilled with TSG6- or IL-4–treated MФs showed significantly reduced lung vascular permeability; *n* = 6 mice per group. *P < 0.05 vs. UT MФs group. (*C*) Ly6G population was measured by flow cytometric analysis in BALF of mice i.t.-instilled with either UT- or TSG6-treated BMMФs, and challenged with i.p. LPS (10 mg/kg) for 24 h. (*D*) BALF concentrations of proinflammatory (TNF-α, IL-6, IL-12, and IFN-γ) and antiinflammatory (IL-10 and IL-4) cytokines measured with ELISA. *P < 0.05 vs. UT MФs group.

We next determined whether MФ phenotype transition is sufficient to resolve LPS-induced inflammatory lung injury. Mice were challenged with a lethal dose of LPS (20 mg/kg i.p.) and simultaneously i.t.-instilled with BMMФs treated and not treated with TSG6. We observed 100% mortality in mice challenged with LPS plus i.t.-instilled with control BMMФs (Fig. 4*A*), whereas LPS-challenged mice receiving i.t. instillation of BMMФs primed with TSG6 or IL-4 showed markedly reduced mortality (Fig. 4*A*). In other experiments, we determined the effects of TSG6-primed BMMФs on resolution of LPS-induced lung injury. We observed that i.t. instillation of TSG6-primed BMMФs significantly reduced lung vascular permeability (Fig. 4*B*), PMN accumulation in BALF (Fig. 4*C*), and generation of inflammatory cytokines in BALF (Fig. 4*D*).

## Discussion

Dead PMNs prolong inflammation and fail to restore tissue homeostasis through release of chemokines, chemoattractants, and tissue debris (17). Therefore, effective clearance of apoptotic PMNs via efferocytosis by AMФs, an immunologically silent process, is a prerequisite for resolution of inflammation and tissue injury (18, 19). Studies showed that MФ phenotype transition from inflammatory to antiinflammatory cells terminates inflammatory signals in MФs (9, 10, 20). Efferocytosis is mediated by TAM receptors in phagocytes such as AMФs and the highly conserved recognition “eat me” signaling molecule phosphatidyserine present on target cells (2, 11). The recognition of dead cells removed by efferocytosis is distinct from the recognition of foreign materials by innate and adaptive immune systems (2, 21). Although clearing of dead cells, cell fragments, and other effete material through phagocytosis is essential for resolution of inflammation (1, 3, 11), the mechanistic links between MФ phenotype transition and activation of efferocytosis are unclear.

Here, utilizing the murine model of PMN-dependent ALI induced by LPS (22, 23), we observed (*i*) the temporal transition of AMФs to an antiinflammatory phenotype coupled with generation of the cytokines IL-4 and TSG6, (*ii*) increases in AMФs expressing antiinflammatory markers indicative of phenotype shift in AMФs, and (*iii*) enhanced expression of the efferocytotic ligand Gas6 inducing the activation of efferocytosis machinery in AMФs. These findings are consistent with the MФ phenotype shift occurring in association with activation of efferocytosis. We focused on Gas6, a central component of efferocytosis on the MФ surface. The binding of Gas6 to TAM receptors expressed on the MФ membrane bridges with the phosphatidylserine signal expressed on apoptotic PMN membrane (6, 8). We identified the requisite role of STAT6-mediated expression of Gas6 in response to IL-4 and TSG6 signaling as a central mechanism of activation of efferocytosis in AMФs.

MФs play a key role in resolving inflammation through their ability to engulf bacteria and dead cells (1, 21). MФs are also plastic in that they adopt a proinflammatory or antiinflammatory property (9, 10). This shift depends on the microenvironment (or niche) and release of specific cytokines such as IL-4 and TSG6 (3, 14), which we used in the present study to address the role of MФ shift in efferocytosis. Studies showed that efferocytosis can itself lead to MФ antiinflammatory phenotype transition, as evidenced by the release of antiinflammatory and proresolving mediators IL-10 and TGF-β (2, 24–26). In this context, IL-13 (a MФ antiinflammatory transition cytokine) generated by regulatory T cells stimulated IL-10 production in MФs, which in turn induced efferocytosis and resolution of inflammation. Studies also showed that engagement of the efferocytosis machinery is only one feature of a broader antiinflammatory phenotype shift in MФs (11, 27).

The transcription factor STAT6 induces MФ phenotype transition in response to IL-4 and IL-13 signaling (28, 29). IL-4 binding to IL-4 receptor-α phosphorylates STAT6, resulting in its dimerization and translocation to the nucleus and triggering expression of target genes (12, 29). Activation of STAT6 was shown to repress transcription of inflammatory genes and resolve inflammation (13, 30). This was evident in studies in which IL-4 administration via i.p. injection accelerated resolution of lung inflammation in a STAT6-dependent manner (3). In the present study, we found that STAT6 binding to the Gas6 promoter induced the expression of Gas6 in AMФs. Release of cytokines is a likely cause of STAT6 activation (28, 31). We observed the generation of IL-4 and TSG6 in BALF of LPS-challenged mice, both TSG6- and IL-4–induced STAT6 activation, and transcription of Gas6. Suppression of STAT6 abrogated both Gas6 expression and efferocytosis of dead PMNs, demonstrating that the antiinflammatory MФ phenotype inducing STAT6 plays a vital role in mediating efferocytosis through Gas6 expression.

We investigated whether efferocytosis in the endotoxemia model is essential for resolution of inflammatory lung injury. We observed that adoptive transfer of BMMФs primed with STAT6-activating mediators in which Gas6 was induced effectively cleared apoptotic airspace PMNs, suppressed production of inflammatory cytokines and lung vascular leaking in endotoxemic mice, and significantly reduced mortality. The protective effects of phenotype-shifted BMMФs required the expression of Gas6, indicating the necessity of priming the MФs and intact efferocytotic machinery for resolution of inflammation. We cannot, however, rule out the possibility that the adoptively transferred BMMФs may have in some way affected resident AMФs, which could account for the protection. Nevertheless, our findings raise the intriguing possibility of MФ-based therapy through instillation of the shifted MФs in restoring homeostasis in ALI patients through activation of efferocytosis of PMNs in the airspace.

## Materials and Methods

The detailed methods are discussed in the *SI Appendix*.

### Mouse Experiments.

Male and female C57BL/6 mice aged 8–10 wk (Jackson Laboratory) were used for experiments. Mice were bred and maintained under specific pathogen-free conditions at the University of Illinois at Chicago animal facility, and all protocols were approved by the Animal Care Committee administered through the Office of Animal Care and Institutional Biosafety.

### Collection of BALF and Cytokine ELISAs.

Bronchoalveolar lavage fluid (BALF) was collected from mice. The supernatant was used for the measurement of cytokines with an ELISA kit (eBiosciences), and the pelleted cells were stained with Ly6G (127628; Biolegend) and subjected to flow cytometry analysis.

### Isolation of AMФs.

AMФs were isolated from BALF as described previously (32).

### Bone Marrow–Derived Macrophages (BMMФ).

Bone marrow cells were isolated from mouse femur and tibial cavities and incubated at ∼2 × 10^6^ cells/mL in DMEM supplemented with 10% (vol/vol) FBS, 1% (vol/vol) streptomycin/penicillin, and 10% (vol/vol) L929-conditioned media for 6 d. Cells were used for experiments on day 6 of culture. MФs were labeled with CellTracker CMTPX red dye for 15 min (C34552, Thermo Fisher Scientific) and used for experiments.

### Isolation of PMNs and Induction of Apoptosis.

PMNs were isolated from bone marrow cells by density-gradient centrifugation using Histopaque as described previously (33). Isolated PMNs were exposed to UV irradiation (254 nm, UVS-26, 6-W bulb, 0.02 J/s/cm^2^) for 15 min to induce apoptosis and then incubated at 37 °C for 4 h in an incubator containing 5% CO_2_ (15).

### Adoptive Transfer of BMMФs in Mice.

BMMФs were stimulated with 400 ng/mL rTSG6 (2104-TS-050; R&D Systems) or 100 ng/mL rIL-4 (214-14; Peprotech) for 6 h in culture conditions in vitro, and adoptively transferred by noninvasive i.t. instillation into mice. For the induction of lung injury, mice received a single dose of LPS i.p. (10 mg/kg body weight; *Escherichia coli* strain 0111: B4; Sigma-Aldrich).

### Immunostaining.

BMMФs were grown on coverslips and stimulated with TSG6 (400 ng/mL) for 6 h. After 4 h of TSG6 treatment, MФs were overlaid with apoptotic PMNs (1:10 ratio) labeled with CellTracker CMTPX red dye for 15 min for 2 h. After vigorous washing with PBS and fixation with acetone-methanol, MФs engulfing apoptotic PMNs were analyzed by confocal microscopy (LSM 880; Zeiss).

### In Vivo Imaging of Lungs.

The surgical methods for gaining access to lungs were based on Looney et al. (34). BMMФs stimulated with rTSG6 (TSG6 MФs) or rIL-4 (IL-4 MФs) were labeled with CellTracker CMTPX red dye for 15 min as described previously and adoptively transferred into mice. An Ultima resonant-scanning 2-photon microscope (Bruker) with an Olympus XLUMPlanFL N 20× (NA 1.00) was used to collect dual-color images (emission filter: 460/50 nm for Brilliant Violet 421, 525/50 nm for Alexa 488, and 595/50 for CMTPX) with 820 nm excitation.

### Promoter Analysis and ChIP Assay.

Consensus binding sites for transcription factor STAT6 in the 5′-regulatory region of the Gas6 gene were analyzed with the Eukaryotic Promoter Database (SIB). ChIP assays were done as described previously (35).

### RNA Extraction and qRT-PCR.

Total RNA isolated from BMMФs were reverse-transcribed for the synthesis of cDNA according to manufacturer’s instructions (K1612; Thermo Fisher Scientific,). The cDNA obtained was mixed with SYBR Green PCR Master Mix (Applied Biosystems), and gene-specific primers were used for PCR. An ABI Prism 7000 system was used for quantitative PCR analysis.

### Immunoblotting.

MФs were lysed in RIPA buffer containing protease and phosphatase inhibitor mixture. Cell lysates were centrifuged at 20,000 × *g* for 15 min at 4 °C, and cleared supernatant was used for immunoblotting.

### Transfection with siRNA.

BMMФs were transfected with control (sc-37007) or two independent Gas6 siRNAs (sc-35451; Santa Cruz Biotechnology; s66469; Life Technologies) for 36 h using Lipofectamine 3000 reagent (L3000015; Thermo Fisher Scientific) in 2 mL growth medium according to the manufacturer’s instructions. For the assessment of transfection efficiency, immunoblotting was done as described earlier.

### Measurement of Lung Vascular Permeability.

Lung vascular permeability was measured as described previously (14).

## Supplementary Material

Supplementary File
