# Supplementary material for: STAT6 induces expression of Gas6 in macrophages to clear apoptotic neutrophils and resolve inflammation
Source: Proc Natl Acad Sci U S A. 2019 Jul 30;116(33):16513–8. doi: 10.1073/pnas.1821601116 (PMC6697797; doi:10.1073/pnas.1821601116)
Supplement: Supplementary File [file pnas.1821601116.sapp.pdf]

STAT6 Induces Expression of Gas6 in Macrophages to Clear Apoptotic Neutrophils and Resolve Inflammation

Saroj Nepal[a], Chinnaswamy Tiruppathi[a], Yoshikazu Tsukasaki[a], Joseph Farahany[a], Manish Mittal[a], Jalees Rehman[a], Darwin J. Prockop[b,], and Asrar B. Malik[a, 1]

[a]Department of Pharmacology and Center for Lung and Vascular Biology, University of Illinois College of Medicine, Chicago, IL 60612 and [b]Institute for Regenerative Medicine, Texas A & M University Health Science Center and College of Medicine, Bryan, TX 77807

To whom correspondence may be addressed: Email: abmalik@uic.edu or Prockop@medicine.tamhsc.edu

Classification: Medical Sciences, Biological Sciences, Immunology and Inflammation

Key words: macrophage, STAT6, Gas6

Running title: STAT6 induces Gas6 expression in macrophages

www.pnas.org/cgi/doi/10.1073/pnas.1821601116

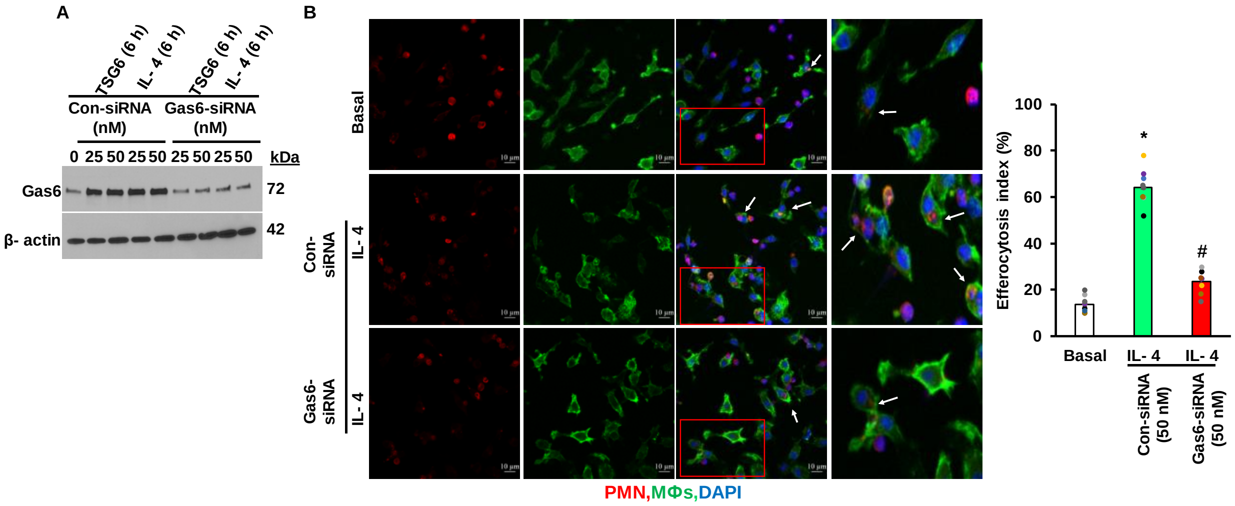
A
TSG6 (6 h)
IL- 4 (6 h)
TSG6 (6 h)
IL- 4 (6 h)
Con-siRNA (nM)
Gas6-siRNA (nM)
0 25 50 25 50 25 50 25 50
kDa
Gas6
72
β- actin
42
B
Basal
Con-siRNA
IL- 4
Gas6-siRNA
IL- 4
PMN, MΦs, DAPI
Efferocytosis index (%)
100
80
60
40
20
0
*
#
Basal
IL- 4
IL- 4
Con-siRNA (50 nM)
Gas6-siRNA (50 nM)

Supplemental fig. 1

(A) BMMΦs transfected with indicated nM of control or Gas6-siRNA (s66469) were used to determine Gas6 protein expression by immunoblot. (B)As described in fig. 1F, confocal analysis was performed to assess efferocytosis by BMMΦs transfected with control or Gas6-siRNA (s66469), and treated with IL- 4 (100ng/ml, 6h). F4/80 antibody (green) BMMΦs; apoptotic PMNs (red). The quantified data from three different experiments are shown in bar graph. *P< 0.05 vs control.

Materials and methods

Mouse experiments.

All mice were housed at the University of Illinois Animal Care Facility in accordance with institutional and NIH guidelines. Veterinary care of animals and related animal experiments were approved by the institution. Male and female C57BL/6 mice aged 8-10 weeks obtained from Jackson Laboratory were used for experiments.

Collection of bronchoalveolar lavage Fluid (BALF) and cytokine ELISAs.

For the collection of BALF, the mice were anesthetized by ketamine/xylazine (45 mg/kg and 8 mg/kg, respectively) and the upper part of the trachea was cannulated, and the lung was lavaged thrice with 1 ml of HBSS supplemented with 0.4 mM EDTA and protease inhibitor mixture. BALF was centrifuged at 700 × g for 5 min at 4°C. The collected supernatant was used for the measurement of cytokines through ELISA kit (ebiosciences) as per manufacturer's instructions. The pelleted cells were stained with Ly6G (127628; Biolegend) and subjected to flow cytometry analysis. Mouse Gas6 ELISA Kit (ab155447) was obtained from Abcam and TSG6 ELISA kit (008859) was purchased from Ray Biotech.

Isolation of alveolar macrophages (AMΦs).

AMΦs were isolated from BALF as described previously (1). Briefly, BALF was centrifuged at 700 × g for 5 min at 4°C. The cells were then incubated overnight at 37°C. The cells adhering to the bottom of dish were collected and used for experiments. The purity of isolated AMΦs was determined using fluorescently labeled antibodies recognizing surface antigens F4/80 and CD11b.

Bone marrow-derived macrophages (BMMΦ).

For the isolation of bone marrow cells, femur and tibial cavities from mice were flushed, and the marrow cells were passed through 70 µm cell strainer followed by centrifugation at 500 g for 10

min and incubated at approximately 2×10^6 cells/mL in DMEM supplemented with 10% (vol/vol) FBS, 1% (vol/vol) streptomycin/penicillin, and 10% (vol/vol) L929-conditioned media for 6 days. Cells were used for experiments at day 6 of culture. MΦs were labelled with Cell Tracker Red CMTPX Dye for 15 min (C34552, Thermofisher Scientific) and used for experiments.

Isolation of PMNs and induction of apoptosis.

PMNs were isolated from the bone marrow cells by density-gradient based centrifugation using Histopaque as described previously (2). Isolated PMNs were exposed to UV irradiation (254 nm, UVS-26, 6-W bulb, 0.02J/s/cm^2) for 15 min to induce apoptosis and then incubated for 4 h in an incubator at 37°C containing 5% CO_2 (3). Apoptosis induction was analyzed by flow cytometry after staining with FITC Annexin V (640905; Biolegend), and Propidium Iodide (421301; Biolegend). Approximately 90% of PMNs were apoptotic.

Adoptive transfer of BMMΦs in mice.

BMMΦs were stimulated with rTSG6 400 ng/ml (2104-TS-050; R&D Systems) or rIL-4 100 ng/ml (214-14; Peprotech) for 6 h in culture conditions *in vitro*. These stimulated BMMΦs were then adoptively transferred by noninvasive i.t. instillation into mice. For i.t. instillation, the mice were anesthetized by ketamine/xylazine (45 mg/kg and 8 mg/kg, respectively) and were suspended on a flat board and placed in a semirecumbent position with the ventral surface and rostrum facing up. Using a curved blade forceps, the tongue was gently and partially retracted rostrally, and 50 µl containing 2×10^6 cells was placed in the trachea. For the induction of lung injury, mice received a single dose of LPS i.p. (10 mg/kg body weight; Escherichia coli strain 0111: B4; Sigma-Aldrich) and concurrently BMMΦs i.t. instilled, and lungs were collected after 24 h for the determination of Evans blue dye conjugated with albumin (EBA) uptake. For survival studies, mice were injected i.p. with 20 mg/kg LPS and monitored four times daily for 72 h.

Immunostaining.

BMMΦs were grown on coverslips and stimulated with TSG6 (400 ng/ml) for 6 h. After 4 h of TSG6 treatment, MΦs were overlaid with apoptotic PMNs (1:10 ratio) labelled with CellTracker Red CMTPX Dye for 15 min (C34552, Thermofisher Scientific) for 2h. After vigorous washing with PBS and fixation with acetone-methanol, MΦs engulfing apoptotic PMNs were analyzed by Confocal microscopy (Zeiss LSM 880). Experiments were done in triplicate and the efferocytosis index was expressed as the percentage of MΦs containing at least one ingested PMN obtained from 10 different field of view.

***In vivo* imaging of lungs.**

The surgical methods for gaining access to lungs are based on Looney *et al* (4). BMMΦs stimulated with rTSG6 (TSG6 MΦs) or rIL-4(IL-4 MΦs) were labelled with Cell Tracker Red CMTPX Dye for 15 min as described previously and adoptively transferred into mice. As described above, mice received a single i.p. dose of LPS (10 mg/kg body weight) and concurrently BMMΦs (2 x 10^6 cells) i.t. instilled for 24 h. Before the surgery, retro orbital i.v. injections of Brilliant Violet 421-labeled LY6G antibody (10 µg/mice) (clone 1A8, Biolegend) and Alexa 488 -labeled CD31 antibody (20 µg/mice) (clone 390, Biolegend) were made to stain PMNs and lung vessels, respectively. A resonant-scanning two-photon microscope (Ultima Multiphoton Microscopes, Bruker) with an Olympus XLUMPlanFL N 20x (NA 1.00) was used to collect dual-color images (Emission filter; 460/50 nm for Brilliant Violet 421, 525/50 nm for Alexa 488 and 595/50 for CMTPX) with 820 nm excitation. Images were processed and analyzed by image J and customized LabVIEW programs. The PMN density was quantified with Image J by measuring fluorescent intensities of PMNs in microvessels and alveoli. The alveoli were manually outlined and fluorescent intensity signal of PMNs in each outlined region was measured and normalized to

basal condition in wild-type mice with unstimulated macrophage. The macrophages present in the alveolar space were quantified based on counting cell number in each field of view.

Promoter analysis and ChIP assay.

Consensus binding sites for transcription factor STAT6 in 5′ regulatory region of Gas6 gene was analyzed with Eukaryotic promoter database (SIB, Switzerland). ChIP assays were done as described previously (5). STAT6 ChIP antibody (67906; Biolegend) was used for immunoprecipitation. The following primer pairs were used for determining STAT6 binding to to the mGas6 gene: STAT6 (SB1) forward, 5′-CATTCCAAGCACATGGTGTC -3′, and reverse, 5′-GTTTGAAGGTGGGGCTAACA-3′ (-81 to -295); STAT6 (SB2) forward, 5′-CCAAGAATCTGGGTTGCATT -3′, and reverse 5′- CCAAAGCTTTCTGGCTCTTG-3′ (-341 to -582); STAT6 (SB3) forward, 5′-TGCCCAAGAATGAAGATTCC -3′, and reverse 5′-GGTGGGGCCTAGTGTGAGTA- 3′ (-729 to -949); STAT6 (SB4) forward, 5′-CCCCGCCCTACTCACACTA -3′, and reverse 5′- CTCCGGTCATCCTGGCTAAG-3″ (-921 to -1077); STAT6 (SB5) forward, 5′-TGGCTTCCGAGTCTTCTCAC -3′, and reverse 5′-CGTAATGCCTGTGTGTCTGG-3 (-1228 to -1422). The DNA-protein interaction was calculated with the following formula: $2^{\Delta Ctx} - 2^{\Delta Ctb}$, where ΔCtx = the cycling threshold of input DNA – the cycling threshold of sample DNA, and ΔCtb = the cycling threshold of input DNA – the cycling threshold of control antibody.

RNA extraction and qRT-PCR.

Total RNA isolated from BMMΦs were reverse-transcribed for the synthesis of cDNA according to manufacturer’s instructions (Thermofisher Scientific, K1612). The cDNA obtained was mixed with SYBR Green PCR Master Mix (Applied Biosystems) and gene-specific primers were used for PCR. An ABI Prism 7000 was used for quantitative PCR analysis. The primer sequences used

for amplification of target genes were as follows: Gas6 forward, 5′-TTAGCCAGGATGACCGGAG-3′ and Gas6 reverse, 5′-ACAGCACAGTGTGAGAAGACTC-3′; CYP forward, 5′-CTTGTCCATGGCAAATGCTG-3′ and CYP reverse, 5′-GTGATCTTCTTGCTGGTCTTGC-3′. Comparative threshold (Ct) method was used for the determination of target mRNA after normalizing target mRNA Ct values to those for Cyclophilin CYP (ΔCt).

Immunoblotting.

MΦs were lysed in RIPA buffer (50 mM Tris-HCl, pH 7.5, 150 mM NaCl, 1 mM EGTA, 1% Triton X-100, 0.25% sodium deoxycholate, and 0.1% SDS) containing protease and phosphatase -inhibitor cocktail. Cell lysates were centrifuged at 20,000 × g for 15 min at 4°C, and cleared supernatant was used for immunoblotting. For immunoblotting, 50-75 μg of solubilized proteins were loaded and resolved by 4–15% gradient separating gel under reducing conditions. The proteins were transferred to PVDF membranes, blocked with 5% dry milk, incubated with the designated primary antibodies overnight, washed and incubated with the appropriate horseradish peroxidase-conjugated secondary antibody. Protein bands were detected by enhance chemiluminescence. The membranes were then stripped and reprobed with β-actin antibody as the loading control. The following antibodies were used for immunoblotting: arginase-1 (sc-20150; Santacruz Biotechnology), CD206 (AF2535; R&D Systems), Gas6 (AF986; R&D Systems), pSTAT6^{Y641} (9361S; cell signaling technology), Total STAT6 (51-9002106; BD biosciences), and anti–β-actin (A5316; Sigma-Aldrich). Band intensity was determined by densitometry with the aid of ImageJ software.

Transfection with small interfering RNA (siRNA)

BMMΦs were transfected with control (sc-37007) or two independent Gas6-siRNAs (sc-35451, Santa Cruz Biotechnology and s66469, Life technologies), for 36 h using Lipofectamine 3000 reagent (L3000015; Thermofisher Scientific) in 2 ml growth medium according to the manufacturer's instructions. For the assessment of transfection efficiency, immunoblotting was done as described earlier.

Measurement of lung vascular permeability.

Lung vascular permeability was measured as described previously (6). Mice were anesthetized and injected retro-orbitally with 150 μl of EBA (30 mg/kg). After 30 min of EBA injection, mice were sacrificed, and lung tissue was harvested for EBA measurement.

1. Wu J, *et al.* (2013) Activation of NLRP3 inflammasome in alveolar macrophages contributes to mechanical stretch-induced lung inflammation and injury. *J Immunol.* 190(7):3590-3599. doi: 3510.4049/jimmunol.1200860. Epub 1202013 Feb 1200822.
2. Swamydas M, Luo Y, Dorf ME, & Lionakis MS (2015) Isolation of Mouse Neutrophils. *Curr Protoc Immunol.* 110:3.20.1-3.20.15.(doi):10.1002/0471142735.im0471140320s0471142110.
3. Jiang C, *et al.* (2017) Inactivation of Rab11a GTPase in Macrophages Facilitates Phagocytosis of Apoptotic Neutrophils. *J Immunol.* 198(4):1660-1672. doi: 1610.4049/jimmunol.1601495. Epub 1602017 Jan 1601494.
4. Looney MR, *et al.* (2011) Stabilized imaging of immune surveillance in the mouse lung. *Nat Methods.* 8(1):91-96. doi: 10.1038/nmeth.1543. Epub 2010 Dec 1012.
5. DebRoy A, *et al.* (2014) Cooperative signaling via transcription factors NF-kappaB and AP1/c-Fos mediates endothelial cell STIM1 expression and hyperpermeability in response to endotoxin. *J Biol Chem.* 289(35):24188-24201. doi: 24110.21074/jbc.M24114.570051. Epub 572014 Jul 570011.
6. Mittal M, *et al.* (2016) TNFalpha-stimulated gene-6 (TSG6) activates macrophage phenotype transition to prevent inflammatory lung injury. *Proc Natl Acad Sci U S A.* 113(50):E8151-E8158. doi: 8110.1073/pnas.1614935113. Epub 1614932016 Nov 1614935128.
